# An approach for diagnosing plasma cell myeloma by three-color flow cytometry based on kappa/lambda ratios of CD38-gated CD138^+^ cells

**DOI:** 10.1186/1746-1596-7-131

**Published:** 2012-09-28

**Authors:** Shoko Nakayama, Taiji Yokote, Yuji Hirata, Kazuki Iwaki, Toshikazu Akioka, Takuji Miyoshi, Ayami Takayama, Uta Nishiwaki, Yuki Masuda, Toshiyuki Ikemoto, Hidema Tanaka, Yasuichiro Nishimura, Motomu Tsuji, Toshiaki Hanafusa

**Affiliations:** 1 First Department of Internal Medicine (I), Osaka Medical College, 2-7 Daigakumachi, Takatsuki City, Osaka, 569-0801, Japan; 2 Central Clinical Laboratory, Osaka Medical College, Takatsuki City, Osaka, Japan; 3 Division of Mathematics, Osaka Medical College, Takatsuki City, Osaka, Japan; 4 Division of Surgical Pathology, Osaka Medical College, Takatsuki City, Osaka, Japan

**Keywords:** Plasma cell myeloma, Diagnosis, kappa/lambda ratio, CD138, CD38

## Abstract

**Background:**

World Health Organization (WHO) criteria are commonly used to diagnose plasma cell myeloma (PCM); however, these criteria are complex and require several laboratory parameters. For differentiating reactive plasmacytosis from clonal plasma cell (PC) neoplasms such as PCM, it is important to accurately determine the expression of cytoplasmic immunoglobulin light chains.

**Methods:**

We retrospectively analyzed the records of 27 selected patients with PCM who underwent bone biopsies for confirmative diagnosis according to WHO criteria. Twenty-three controls were also investigated. In the present study, all the samples were analyzed using flow cytometry (FC) in the side scatter vs. CD38 histogram mode, and the CD38-gated PC population was identified. Bivariate histograms of CD138/kappa and CD138/lambda were assessed, and the ratios of dual-positive cells to the CD138^+^ PC population were calculated. The kappa/lambda ratio was defined as the ratio of CD138/kappa to CD138/lambda.

**Results:**

PCM cells were distinguished from normal PCs using cutoff levels between 0.76 and 1.5, at a sensitivity of 96.3% and specificity of 95.7%.

**Conclusions:**

Three-color FC analysis is simple to perform and inexpensive, with clinically relevant data obtained soon after the completion of FC measurements. The detection of the cytoplasmic kappa/lambda ratio of CD38-gated CD138^+^ PCs may be a useful tool in the diagnosis of PCM. To the best of our knowledge, this report represents the first diagnostic assessment of the cytoplasmic kappa/lambda ratio in CD38-gated CD138+ PCs using FC analysis. This method may help in more simple, efficient, rapid, and accurate diagnosis of PCM.

**Virtual slides:**

The virtual slide(s) for this article can be found here: http://www.diagnosticpathology.diagnomx.eu/vs/1568085959771735

## Introduction

Plasma cell myeloma (PCM) is a bone marrow (BM)-based, multifocal plasma cell (PC) neoplasm associated with the presence of M protein in serum and/or urine. Most cases involve disseminated tumor cells in BM. World Health Organization (WHO) criteria are usually employed to diagnose PCM [1]. These criteria are based on a combination of pathological, radiological, and clinical features. However, the diagnostic criteria are complex, and several laboratory parameters are required for an accurate diagnosis. The diagnosis involves evaluating the clinical burden of PC infiltration, analysis of radiologically detectable bone lesions, electrophoretic determination of monoclonal immunoglobulins (Igs), and assessment of PCs in BM. Using retrospective analyses, we investigated the diagnostic value of an abnormal cytoplasmic kappa/lambda ratio of CD38-gated CD138^+^ PCs based on flow cytometry (FC) assessment. We determined the diagnostic range for the kappa/lambda ratio that maximized the diagnostic sensitivity and specificity and minimized false-positive and false-negative results.

## Materials and methods

### Case and control samples

We retrospectively analyzed the records of 27 patients with PCM who underwent bone biopsies between 2000 and 2010 at the Osaka Medical College, Osaka, Japan. Final diagnosis of PCM was established according to WHO criteria. We obtained written consent from each patient. Eleven males and 16 females whose ages ranged from 47 to 85 years [mean ± standard deviation (SD), 66.2 ± 10.1 years] were included. Monoclonal components of the myelomas in the patient group were IgA type (n = 5), IgG type (n = 16), IgM type (n = 2), Bence-Jones type (n = 3), and nonsecretory type (n = 1); monoclonal components of the Ig light chain (LC) were kappa type (n = 9) and lambda type (n = 17).

For the control group, BM samples were obtained and examined from 23 patients who were recovering from inflammatory disorders and healthy people who were initially suspected as having hematological diseases and underwent examination for the same. (12 males and 11 females; age range, 29–81 years; mean ± SD, 62.5 ± 12.9 years). The monoclonal components in their serum and urine were undetectable by immunofixation electrophoresis.

### Sample preparation and FC analysis

For preparation of the cell suspensions, BM samples were collected in EDTA anticoagulant. All the samples were washed twice in phosphate-buffered saline (PBS) and resuspended in PBS at a concentration of approximately 2 × 10^6^ cells/ml. The cells were resuspended in 100 μl of Reagent 1 (fixation) (IntraPrep™; Beckman Coulter, Brea, CA, USA) for 15 min at room temperature and washed in PBS. After washing, permeabilization was induced by incubation in 100 μl of Reagent 2 (permeability), with the remaining erythrocytes being lysed simultaneously. The samples were stained for 30 min at 4°C with either phycoerythrin cyanine 5.1 (PC5)-conjugated CD38 (J.33; Beckman Coulter), phycoerythrin-conjugated CD138 (HIB19; Becton Dickinson, San Jose, CA, USA), fluorescein isothiocyanate (FITC)-conjugated kappa [goat F(ab^′^)2 anti-human kappa; Biosource, Camarillo, CA, USA], or lambda [goat F(ab^′^)2 anti-human lambda; BioSource Corporation, Bethesda, MD, USA]. Negative control samples were stained for 30 min at 4°C with FITC-conjugated rat antihuman Ig (LODNP1; Coulter) and PC5-conjugated rat antihuman Ig (LODNP1; Coulter) (Table 1). All the samples were rinsed twice in PBS and analyzed on a flow cytometer (EPICS XL; Coulter) using System II software, version 3.0 (Coulter). Approximately 10,000 cells were analyzed.

**Table 1:** List of antibodies/probes

**Antibody**	**Probe**	**Source**
CD38	J.33	Becton Dickinson, San Jose, CA, USA
CD138	HIB19	Becton Dickinson, San Jose, CA, USA
Kappa	goat F(ab’)2 anti-human kappa	BioSource Corporation, Bethesda, MD, USA
Lambda	goat F(ab’)2 anti-human lambda	BioSource Corporation, Bethesda, MD, USA
Immunoglobulin	LODNP1	Becton Dickinson, San Jose, CA, USA

**Figure 1:**
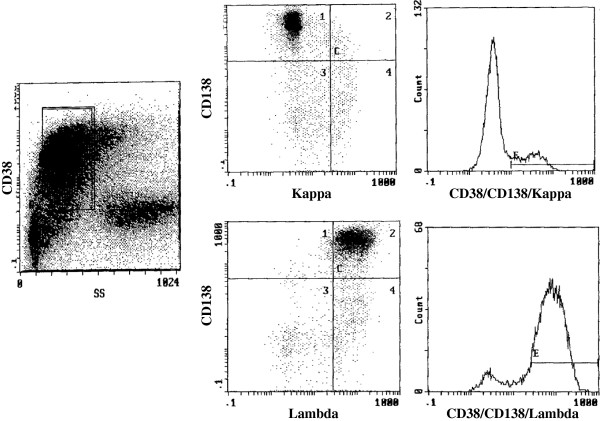
**Three-color flow cytometry analysis.** Side scatter vs. CD38 histogram mode with CD38 gating (left). Bivariate histogram of CD138 (phycoerythrin) and kappa or lambda (fluorescein isothiocyanate) (right).

The first step was to exclude any debris and fat cells on the forward scatter (FSC)/side scatter (SSC) histograms. All the samples were analyzed in the SSC vs. CD38 histogram mode with the CD38-gated PC population being identified by its characteristic bright CD38 expression. Following this, CD138/kappa and CD138/lambda were assessed on bivariate histograms, and the ratios of dual-positive cells to the CD138^+^ PC population were calculated (Figure 1). The kappa/lambda ratio was defined as the ratio of CD138/kappa to CD138/lambda.

### Statistical analysis

The number of observations, means, SDs, medians, minimums, and maximums for continuous variables as well as the number, percentage, sensitivity, and specificity for categorical data were calculated. All analyses were performed using SPSS software (ver. 18 for Windows; SPSS Japan Inc., Tokyo, Japan). The kappa/lambda ratios of the 23 healthy individuals were limited to a narrow range of 0.76 to 8.80, whereas those of the 27 patients were distributed over a wider range of 0.00102 to 478.5. Thus, by choosing two levels, *l* and *u,* with *l* < *u,* to distinguish the patients from controls, we could create a frequency table with two rows and three columns (Table 2) and define (n_d_ + n_b_ + n_f_)/50 as the “error rate” for disease diagnosis using the kappa/lambda ratio. We decided that the values for both the lower and upper cutoff levels would be those with the lowest error rate among the error rates of 5000 frequency tables created by moving *l* (0–0.99 by 0.01) and *u* (1.1–6.0 by 0.1), independently. We also computed the sensitivity and specificity of these cutoff values. All the procedures were performed using R statistical software ( http://www.Rproject.org). R provides a wide variety of statistical analyses and is highly extensible. R is designed around a true computer language and allows users to add additional functionality by defining new functions.

**Table 2:** Frequency table in statistical analyses

	***x*** **<** ***l***	***l ≤ x ≤*** ***u***	***u < x***	**Total**
**Plasma cell myeloma**	**na**	**nb**	**nc**	**27**
**Control**	**nd**	**ne**	**nf**	**23**

## Results

The positive cell ratios were 52.7% − 99.4% (mean ± SD, 82.7% ± 20.5%) for kappa and 0.20% − 52.2% (mean ± SD, 16.1% ± 20.1%) for lambda in the patients with kappa-type PCM (n = 9). The positive cell ratios were 0.1% − 24.5% (mean ± SD, 5.4% ± 6.5%) for kappa and 67.7% − 99.6% (mean ± SD, 94.2% ± 7.7%) for lambda in the patients with lambda-type PCM (n = 17). The positive cell ratios were 6.5% for kappa and 96.6% for lambda in the patient with nonsecretory-type PCM (n = 1). The range of the kappa/lambda ratio was 1.00 − 478.5 (mean ± SD, 84.20 ± 156.84) in the patients with kappa-type PCM (n = 9), 0.00102 − 0.361 (mean ± SD, 0.063 ± 0.089) in the patients with lambda-type PCM (n = 17), and 0.067 in the patient with nonsecretory-type PCM (n = 1) (Table 3).

**Table 3:** Patient characteristics

** Plasma cell myeloma type**		**Disease stage**	**Percentage of plasma cell bone marrow infiltratin**	**Kappa/lambda ratio**
IgA	lambda	IIIA	66.1%	0.0107
IgA	lambda	IIIB	28.4%	0.0514
IgA	lambda	IIIA	63.3%	0.1045
IgA	lambda	IIIA	43.5%	0.1747
IgA	kappa	IIA	88.2%	1.526
IgG	lambda	IIB	78.1%	0.00102
IgG	lambda	IIIB	30.3%	0.00703
IgG	lambda	IIIA	59.8%	0.00834
IgG	lambda	IIIA	20.7%	0.00127
IgG	lambda	IIB	66.1%	0.0161
IgG	lambda	IIB	59.9%	0.0115
IgG	lambda	IIIB	32.2%	0.0232
IgG	lambda	IIIA	40.1%	0.0418
IgG	lambda	IIIA	20.2%	0.0430
IgG	lambda	IIIB	20.6%	0.0509
IgG	lambda	IIIA	12.3%	0.0604
IgG	kappa	IIIA	13.4%	1.009
IgG	kappa	IA	37.2%	1.522
IgG	kappa	IA	35.0%	15.16
IgG	kappa	IIIb	20.4%	24.02
IgG	kappa	IIIA	9.8%	478.5
IgM	kappa	IIIA	59.5%	16.74
IgM	kappa	IIIA	10.5%	165.1
Bence-Jones	lambda	IIA	6.1%	0.0944
Bence-Jones	lambda	IIIA	67.0%	0.361
Bence-Jones	kappa	IIA	76.8%	52.66
Non-secretary	lambda	IIIB	41.6%	0.0672

In the controls, the positive cell ratio ranged between 32.4% and 90.2% (mean ± SD, 62.5% ± 12.9%) for kappa and between 10.2% and 56.2% (mean ± SD, 43.9% ± 9.0%) for lambda. The range of the kappa/lambda ratio in the controls was 0.76 − 8.80 (mean ± SD, 1.57 ± 1.58).

Among the 27 patients diagnosed with PCM, 26 had kappa/lambda ratios of >1.5 and <0.76. The kappa/lambda ratio was ≥0.76 and ≤1.5 in the remaining patient with PCM. In the 23 controls, 22 had kappa/lambda ratios of ≥0.76 and ≤1.5, whereas the remaining control had a ratio >1.5 (Table 4). We have followed this patient’s clinical course for 5 years, and the patient has remained healthy and does not satisfy WHO criteria for PCM. Thus, PCM cells were distinguished from normal PCs by cutoff levels between 0.76 and 1.5, at a sensitivity of 96.3% and specificity of 95.7%.

**Table 4:** Results of frequency table in statistical analysis

	***x < 0.76***	**0.76** ***≤ x ≤ 1.5***	***1.5 < x***	**Total**
Plasma cell myeloma	18	1	8	27
Control	0	22	1	23

## Discussion

The accurate determination of cytoplasmic Ig (cIg) LC expression is important for differentiating reactive plasmacytosis from clonal PC dysplasia. The former is caused by polyclonal proliferation of PCs, whereas the latter is caused by clonal proliferation of PCs with LC restriction. At present, several methods are available for measuring cIg LCs, including immunohistochemical staining, FC, and immunofluorescent staining [2]. Based on commonly used criteria, LC restriction has been defined as a kappa/lambda ratio >4.0 or <0.5 [3-5]. However, these studies showed cIg LC restriction in patients with B-cell lymphoma and B-cell chronic lymphocytic leukemia but not in those with PCM. One report proposed threshold values for the ratio of cytoplasmic kappa to lambda LCs in PCs for detecting monoclonality in addition to listing criteria for discriminating between PCM and benign reactive plasmacytosis using immunohistological analyses [6]. That study determined the cytoplasmic kappa/lambda ratio in PCs to be 0.4–3.5 for reactive plasmacytosis and ≤0.1 or ≥11.2 for PCM. However, the study did not define the definitive limits of an abnormal kappa/lambda ratio in the diagnostic process. In addition, the PC infiltrate in biopsies and smears was morphologically assessed using only hematoxylin–eosin staining; therefore, the detection of PCs may have been ambiguous.

FC has evolved from a promising new technology to an indispensable, rapid, and sensitive tool for diagnosing hematological malignancies. Many new antibodies, improved gating strategies, and routine use of multiparameter techniques have dramatically improved the diagnostic utility of FC. At present, immunophenotyping is mandatory for the diagnosis and monitoring of acute leukemias and chronic lymphoproliferative disorders. In contrast, for PCM, the use of multiparametric FC in many clinical diagnostic laboratories is currently restricted to clinical research studies and the differential diagnosis of unusual cases [7-9]. PCM has traditionally been difficult to assess by FC, mainly because of the loss of most B lineage-specific antigens and surface Igs during maturation to PCs. A recent study using a multiparameter approach with membrane permeabilization achieved a sensitivity of less than 1% [10]. The generation and identification of markers allows the unequivocal identification of PCs among other hematopoietic cell types such as CD138, and the identification of aberrant PC phenotypes enables to discriminate between normal and neoplastic PCs. The identification of an accurate gating strategy is a critical component of a reproducible and sensitive immunophenotypical assay for analyzing PCs. Various approaches based on CD38, CD138, and/or CD45 expression have been used. The majority of centers report a gating strategy using combined CD38, CD138, and light-scatter characteristics. Notably, there is no formal published consensus method for gating PCs. The percentage of myeloma cells or PCs in BM mononuclear cells based on morphologic examination is almost the same as that of CD38-bright fractions measured by FC. It has therefore been confirmed that normal PCs and myeloma cells are located in the CD38-bright fractions stained with CD38 antibodies [11]. Although CD38 is a common marker expressed on various different cell populations such as T, B, and natural killer cells as well as PCs, high-density expression of CD38 on the cell surface is also a characteristic feature of normal and malignant PCs. CD38 measured as a single marker using FC can therefore help distinguish normal and malignant PCs from other cell types [12]. PCs also typically express weak or absent CD45. The use of two-color analysis with CD38 and CD45 can reliably identify PCs in peripheral blood and BM [13]. Neoplastic PCs are traditionally identified by their CD38^+^ CD45^−/dim^ staining pattern on FC histograms [11,14]. LC restriction has also been defined by this method, with 87% of PCM cases complying with commonly used criteria for B-cell neoplasms (i.e., kappa/lambda ratios >4.0 or <0.5) (2, 3-5). However, it is clear that CD38^+^ CD45^−/dim^ gating may fail to identify myeloma mainly or partly composed of CD45^+^ PCs [15]. In addition, neoplastic PCs typically express CD38 at a lower intensity than normal PCs and may be indistinguishable from contaminating T or B cells [16]. Immature or precursor B lymphocytes are also located in CD38-bright fractions stained with CD38 antibodies, whereas neoplastic PCs may be indistinguishable from immature or precursor B lymphocytes. CD138 (syndecan-1) is a transmembrane heparin sulfate proteoglycan that is typically expressed by PCs and not by T cells, B cells, or immature/precursor B lymphocytes [17]. Assessment of cytoplasmic kappa/lambda expression by FC is important for demonstrating clonality at presentation and is appropriate for the assessment of a stringent complete remission according to IMWG criteria [18]. Standard commercial fixation and permeabilization kits have been reported to be suitable for the assessment of cytoplasmic kappa/lambda detection, and users have not reported any specific advantages or disadvantages of the kits available from different companies. Therefore, the assessment of cytoplasmic kappa/lambda expression by FC is easy to routinize [19]. We attempted to identify PCs using three-color analysis of CD38, CD138, and LCs (kappa and lambda). Next, we investigated the decision limits for an abnormal kappa/lambda ratio of CD38-gated CD138^+^ cells using three-color FC of CD38, CD138, and LCs. By advances in FCM, the identification of aberrant PC phenotypes that enable us to discriminate between normal and neoplastic PCs has allowed us to identify, characterize, and enumerate neoplastic PCs even when few cells are present. There have been no formal studies to identify the minimum requirements for reproducible detection of minimal residual disease, and further investigation is required to identify a common panel. However, based on reported studies, a panel containing CD19 and CD56 would be applicable to at least 90% of patients, with the markers CD20, CD117, CD28, and CD27 likely to increase this applicability to more than 95% of patients. In addition, several participants analyzed CD81 and CD20068 and suggested that these markers should be assessed further [19-23]. Combined assessment of clonality and basic immunophenotype may be useful for screening at diagnosis and follow-up. However, these analyses are more difficult and costly, and it is difficult to routinize these analyses for the screening of newly diagnosed usual PCM in general hospitals.

To the best of our knowledge, our report is the first diagnostic assessment of the cytoplasmic kappa/lambda ratio in CD38-gated CD138^+^ PCs using FC analysis. This study showed that a diagnostic kappa/lambda ratio of <0.76 and >1.5 has a sensitivity of 96.3% and a specificity of 95.7%. Three-color FC analysis is simple to perform and inexpensive, with clinically relevant data obtained soon after completion of the FC measurements. Taking all these factors into consideration, the correct diagnosis of PCM can be achieved more simply, efficiently, and rapidly by this method. This method may improve the accuracy of the diagnostic kappa/lambda ratio determined directly in PCs, and it may be a useful tool for the diagnosis of PCM.

## Abbreviations

PCM: Plasma cell myeloma; BM: Bone marrow; WHO: World Health Organization; PC: Plasma cell; FC: Flow cytometry; FSC: Forward scatter; SSC: Side scatter; PBS: Phosphate-buffered saline; Igs: Immunoglobulins; CIg: Cytoplasmic Ig; LC: Light chain; PC5: Phycoerythrin cyanine 5.1; FITC: Fluorescein isothiocyanate; SD: Standard deviation.

## Competing interests

The authors declare no competing financial interests.

## Authors’ contributions

All authors read and approved the final manuscript.
